# Improved Wavelet Threshold for Image De-noising

**DOI:** 10.3389/fnins.2019.00039

**Published:** 2019-02-08

**Authors:** Yang Zhang, Weifeng Ding, Zhifang Pan, Jing Qin

**Affiliations:** ^1^School of Information and Engineering, The First Affiliated Hospital of WenZhou Medical University, WenZhou Medical University, WenZhou, China; ^2^The Chinese People's Liberation Army 118 Hospital, WenZhou, China; ^3^School of Information and Engineering and Information Technology Centre, WenZhou Medical University, WenZhou, China; ^4^School of Nursing, Hong Kong Polytechnic University, Kowloon, Hong Kong

**Keywords:** wavelet threshold, wavelet transform, image de-noising, MSE, PSNR

## Abstract

With the development of communication technology and network technology, as well as the rising popularity of digital electronic products, an image has become an important carrier of access to outside information. However, images are vulnerable to noise interference during collection, transmission and storage, thereby decreasing image quality. Therefore, image noise reduction processing is necessary to obtain higher-quality images. For the characteristics of its multi-analysis, relativity removal, low entropy, and flexible bases, the wavelet transform has become a powerful tool in the field of image de-noising. The wavelet transform in application mathematics has a rapid development. De-noising methods based on wavelet transform is proposed and achieved with good results, but shortcomings still remain. Traditional threshold functions have some deficiencies in image de-noising. A hard threshold function is discontinuous, whereas a soft threshold function causes constant deviation. To address these shortcomings, a method for removing image noise is proposed in this paper. First, the method decomposes the noise image to determine the wavelet coefficients. Second, the wavelet coefficient is applied on the high-frequency part of the threshold processing by using the improved threshold function. Finally, the de-noised images are obtained to rebuild the images in accordance with the estimation in the wavelet-based conditions. Experiment results show that this method, discussed in this paper, is better than traditional hard threshold de-noising and soft threshold de-noising methods, in terms of objective effects and subjective visual effects.

## Introduction

The transmission, detection and collection of signals are subject to pollution of varying degrees of random noise, influenced by the environment and due to the nature of the work. Thus, the implementation of signal de-noising is necessary. How to filter out the noise in the real signal to obtain effective information, is a current research hotspot. Wavelet transform has a time-frequency local analysis function, and its de-noising results are relatively good. Thus, its application is also very extensive.

In recent years, with the deepening of the intersection and research, along with the application of mathematics and other disciplines, the application of fuzzy mathematics, mathematical morphology, intelligent optimization, neural network, and wavelet theory and technology in image processing, as well as some new methods of noise resistance have emerged. In the early stage, the traditional de-noising method has a low pass filter method, which mainly includes median filtering, linear filtering and adaptive filtering.

During image collection, coding and transmission, all images are visible or invisible to varying degrees of noise. The image noise is divided into three main categories. The first is Gauss noise, which belongs to the category of electronic noise that is produced by a sensitive element caused by the random thermal motion of the electronic components. The second is Poisson noise, which is produced during the process of photoelectric conversion; it has an apparent effect under a weakened light. The third is particle noise, which is produced during the process of photography and can be found under a microscope. The smooth images that can be seen in the photo will display random particle images under the microscope (Auber and Kornprobst, 2006). The purpose of image processing is to perform some operations or processing on the digitized image information, in order to improve the image quality or to achieve a desired effect. For example, the non-uniformity of the sensitivity of sensitive components in photoelectric conversion, transmission error and human factors during the digitization diminishes the quality of an image, which contains various random noises. Sometimes, this random noise will greatly affect the image quality. The noise image affects not only the visual effect of the viewed image, but also affects image processing. Image de-noising aims to retain useful information and reduce or eliminate the interference of noise in the image. De-noising is a key link in image processing. In practical applications, this process is often used as a pretreatment of image processing and recognition, which is the basis of subsequent high-level image processing. Thus far, all studies on image de-noising have focused on this effect and has achieved great progress. However, with the emergence of new problems, people have higher standards of image quality. The traditional image noise removal algorithm is based on the spectrum distribution. In frequency, wavelet de-noising is the commonly used method to separate useful information and noise from images (Johnstone and Silverman, 2005, Othman and Qian, 2006). Other methods include the Markov field model, partial differential equation and LP regularization method (Baske, 2011). This method is also a drawback on regularizing noise. The convergence rate is slow in regions with minimal changes. Sinha and Dougherty (Thomas Asaki and Kevin Vixie, 2010) combined fuzzy mathematics with mathematical morphology and applied it to image processing. In recent years, the feed forward BP neural network was proposed as a filter to de-noise (Noh et al., 2011; Swami et al., 2017). Wavelet transform has also greatly contributed to image de-noising (Michal et al., 2006; Apotsos et al., 2008; Patil, 2015). The correlation coefficient method is based on the correlation between the wavelet coefficients at the corresponding positions for each scale, whereas the noise is neither correlated nor has a weak correlation on each scale to remove the noise. Noise is mainly concentrated in high frequencies, provided that high-frequency processing can achieve the effect of noise reduction. In 2006, Elad and Aharon (2006) proposed a de-noising method on the basis of sparse representation and KSVD dictionary learning. The dictionary learned by the KSVD algorithm (Oey et al., 2013) was used for image de-noising. However, the KSVD algorithm ignores the similarity of the image, and the KSVD algorithm cannot use the detailed information of the image when learning the dictionary on a single scale. At present, the popular multi-scale directional transformation mainly includes: curved wave transformation (Palakkal and Prabhu, 2012), contour wave trans-formation and non-sub sampling contour wave transformation (Amisha et al., 2013). The multi-scale transformation methods can use the inherent geometric features of the natural image data, and all relative wavelet transforms have remarkably improved in direction selection. The 3D block matching algorithm (BM3D) (Lebrun, 2012) is an effective de-noising method for Gauss noise. This algorithm can preserve information such as edge and texture. BM3D comprehensively utilizes non-locality, linear transformation threshold, Wiener filtering, and sparse representation. BM3D also reveals details of different sub-block classes and retains the basic characteristics of each sub-block. This method can improve the resolution in noisy images, however the computation is very large, as each similar block needs to be computed. Pizarro et al. (2010) selected non-local constraints as fidelity items. In similarity measure, the error of noise image and real images was minimal. Moreover, the high-order smoothing of the de-noised image was used as a regularization term, and a non-local data smoothing model was proposed. The model was applied to the similarity between images to obtain a further general model. A selected unsuitable threshold can easily present a Gibbs phenomenon (Huang et al., 2005, Chen et al., 2005). Mallat presented alternating projection (AP) for de-noising. The AP (Mallat and Hwang, 1992; Zhu et al., 2017) method obtains the modulus maxima at each scale after the signal is differentiated on each scale. Then, the non-propagating modulus maxima should restore the signal. The disadvantage of the alternating projection method is that the computation is very large and, the iteration is prone to instability. Li proposed a novel hybrid model based on an extreme learning machine, k-nearest neighbor regression and wavelet de-noising (Li et al., 2017). Using the linear mode to reduce noise will lead to the loss of detail in textured images. The static wavelet transforms (SWT) use time invariance to achieve image de-noising (Wang et al., 2003). Some researchers (Zou et al., 2015; Liu et al., 2017) proposed an approach that searches for candidate matching blocks along the edges that are well-adapted to image details. All similar blocks form a 3D group. De-noising is performed by shrinking the coefficients of the 3D transform applied on these groups. The non-linear diffusion filtering method based on PDE, is a non-linear anisotropic de-noising method (Lee et al., 2005). A non-linear model for de-noising can be excessive in the smoothing of images. Scholars have also studied how to improve the speed of de-noising. The Non-linear Diffusion techniques and PDE-based variational models are very popular in image restoring and processing. The researchers proposed (Fazli et al., 2010; Zeng et al., 2012, 2018) that a heuristic method such as Particle Swarm Optimization (PSO), be used for Complex PDE parameter tuning by minimizing the Structural SIMilarity (SSIM) measure. Tasdizen (2009) enhanced the algorithm efficiency by clustering the blocks with PCA, selecting the similarity between block features as the measurement of block similarity, and optimally estimating the parameters. Mahmoudi and Sapiro (2005) proposed to accelerate the algorithm by eliminating irrelevant neighborhoods in the weighted averaging process. Currently, many researchers have proposed a combination of ways for de-noising. For example, machine learning and random walks are combined with traditional noise removal methods (Huang et al., 2006; Jieru et al., 2016; Liu et al., 2018). Zeng et al. (2017), proposed de-noising and de-blurring gold immune chromatographic strip images via gradient projection algorithms.

Presently, details of images and how to remove noise from them has received increased attention. In this paper, we present an improved threshold to de-noising of MRI images. Experimental results show that the de-noising effect is better than the hard and soft threshold.

## Principle of Wavelet De-Noising Medthod

In current research, there are numerous ways to eliminate noise from images. The application of wavelet de-noising is very extensive. The wavelet method for removing noise has numerous advantages. Not only is the algorithm simple to implement, but it also has a particularly superb effect of de-noising. This method has therefore achieved great results in practical applications. The main principle of wavelet threshold de-noising is based on the strong correlation of the wavelet. The energy concentration of the signal after wavelet transform is often concentrated on the large wavelet coefficient. The noise energy after wavelet transform does not have concentrated characteristics, because the noise does not have the correlation of wavelets. Wavelet coefficients with large amplitude values are mostly signals, whereas the coefficients with small amplitude values are largely noise. The threshold is set on the basis of this property. The hard and soft threshold function method was proposed by Donoho (Donoho, 1995) et al.

The hard threshold is expressed as follows:

(1) $${\hat{w}}_{j,k}=\{\begin{array}{c} w_{j,k},|\;w_{j,k}|\;>=\text{λ}\\ 0,|w_{j,k}|<\text{λ} \end{array}$$

The soft threshold is calculated as follows:

(2) $${\hat{w}}_{j,k}=\{\begin{array}{c} \text{sgn }(w_{j,k})(|w_{j,k}|-\lambda ),|w_{j,k}|\;>=\text{λ}\\ 0,|w_{j,k}|\;<\text{ λ} \end{array}$$

The Semi-threshold function is expressed as follows:

(3) $${\hat{w}}_{j,k}=\{\begin{array}{c} 0,|w_{j,k}|\;<=\text{λ}\\ \text{sgn }(w_{j,k})\frac{{\text{λ}}_{2}(|w_{j,k}|-{\text{λ}}_{1})}{{\text{λ}}_{2}-{\text{λ}}_{1}}\\ w_{j,k},|w_{j,k}|\;>\text{λ} \end{array},{\text{λ}}_{1}\;<\;|w_{j,k}|\;<{\text{λ}}_{2}$$

Although the soft, hard thresholds and semi- thresholds have achieved some results, they all still have drawbacks. The hard threshold function can better preserve boundary information however, the hard threshold function is discontinuous at closed values, thus removing the noise cancellation effect remains rough. Furthermore, its application has some limitations; this function only processes wavelet coefficients smaller than the threshold and does not manage wavelet coefficients larger than the threshold. Therefore, the de-noising result is relatively different. The resulting estimated signal produces additional oscillations. Furthermore, the interference of the noise signal is often mixed in with the wavelet coefficients greater than the closed value function. The soft threshold function has improved overall continuity, and the de-noising result is relatively smooth. However, after noise cancellation, the signal is easily overwhelmed by noise, thereby resulting in difficulties at higher-order derivatives, causing de-noising distortion. Moreover, the soft threshold function performs constant value compression on the wavelet coefficients rather than the threshold. This function directly affects the degree of approximation of the reconstructed signals. The semi-threshold function not only retains a large coefficient, but also has continuity.

The calculation of complexity through this function is higher. In the semi-threshold function, determining the threshold is a difficult point. Therefore, the traditional threshold function has its own defects and has certain limitations in its application, which affects the effect of de-noising.

In this article, we proposed a new threshold function. We improved the threshold to compensate for the deficiency of soft and hard thresholds. In our experiment, we analyzed the experimental results of subjective and objective experiments and concluded that the improved threshold function de-noising effect is better than the hard and soft threshold de-noising.

## Improved Wavelet Threshold De-Noising Method

For the method of threshold de-noising, using hard and soft closed-valued functions, the basic idea is to remove relatively small wavelet coefficients as much as possible. When a hard threshold function is used to de-noise, although it can save the effective part of the original signal relatively well, the reconstructed signal after the noise processing will be very rough. When de-noising with a soft threshold function, the reconstructed signal will easily lose useful signals.

The key to threshold shrinkage is the determination of threshold and threshold functions. If the threshold is selected as large, details will be lost. If the threshold is selected small, then the noise still exists. Although a hard threshold de-noising is simple and easy to implement, it will generate a pseudo-Gibbs phenomenon at the image boundary. In comparison with hard thresholds, soft thresholds are continuous, and the structure of wavelet coefficients is maintained, thereby effectively reducing the pseudo Gibbs phenomenon. However, when wavelet coefficients with an absolute value greater than the threshold value are processed, the image edges will become blurred. To achieve improved results for de-noising, we have enhanced the threshold functions.

### Improved Threshold Functions

The improved threshold function is as follows:

(4) $${\hat{w}}_{j,k}=\{\begin{array}{c} w_{j,k}-\frac{w_{j,k}}{2}.\frac{1}{100^{|w_{j,k}|\text{λ}}-99},|w_{j,k}|\;>\text{λ}\\ \frac{w_{j,k}}{2}.\frac{1}{1-\log_{100}^{|w_{j,k}|/\text{λ}}},|w_{j,k}|\;<=\text{λ} \end{array}$$

The threshold function after adding an adjustment factor is as follows:

(5) $${\hat{w}}_{j,k}=\{\begin{array}{c} w_{j,k}-\frac{w_{j,k}.m}{2}.\frac{1}{100^{|w_{j,k}|\text{λ}}-99},|w_{j,k}|\;>\text{λ}\\ \frac{w_{j,k}.m}{2}.\frac{1}{1-\log_{100}^{|w_{j,k}|/\text{λ}}},|w_{j,k}|\;<=\text{λ} \end{array}$$

Where*m* ∈ *Z*.

When$|w_{j,k}|\overset{}{\underset{}{\to }}\lambda^{+}$, the first inequality of Equation (4) can be written as:

(6) $$\underset{|w_{j,k}|\to \;{\text{λ}}^{+}}{\lim}(\frac{w_{j,k}m}{2}.\frac{1}{100^{|w_{j,k}|\text{λ}}-99})=\frac{\text{λ}}{2}$$

When$|w_{j,k}|\overset{}{\underset{}{\to }}\lambda^{-}$, the second inequality of formula (4) can be written:

(7) $$\underset{|w_{j,k}|\to \;{\text{λ}}^{-}}{\lim}(\frac{w_{j,k}.m}{2}.\frac{1}{1-\log_{100}^{|w_{j,k}|/\text{λ}}})=\frac{\text{λ}}{2}$$

The threshold is continuous at the ±λ point and has high-order derivatives. The threshold function is continuous, and the high order is steerable. The second inequality slowly approaches zero. Here *in* adjusts the shape of the threshold function; *m* adjusts the variation of wavelet coefficient; *k* determines the asymptote of the threshold function. When *k* = 1,we proposed that the threshold function approaches the hard threshold function. When *k* = 0, the threshold function approaches the soft threshold function. Thus, the parameter *k* was adjusted; we proposed that the threshold function can vary between the interval values of soft threshold function and hard threshold function.

The new threshold function proposed in this paper combines the advantages of soft and hard threshold functions. This approach enables the smooth transition of the wavelet threshold curve. The same continuity is achieved in the wavelet domain as the traditional soft threshold function, which improves the shortcomings of hard threshold function discontinuity. Moreover, pseudo-Gibbs phenomenon can be avoided. The new threshold function is a high-order steerable between the intervals of |*w*_*j, k*_| > λ and |*w*_*j, k*_| < = λ . This type of conductivity enables the elimination of the generated oscillation phenomenon in threshold de-noising and the improves the suppression of overkill of the detail coefficient. Thus, the signal after reconstruction can be made smoother.

### Improved Threshold Selection

The threshold is vital in image threshold de-noising, and Donoho (1995) proposed a unified threshold method.

(8) $$\text{λ}=\delta \sqrt{2\log(M*N)}$$

However, this method is not ideal in practical applications and causes over-segmentation (Grace et al., 2000). Through analysis, it was found that the decomposition of the image by wavelet increases with the number of decomposition layers. The energy of noise will become smaller and smaller, and the energy of image information will become increasingly larger. Wavelet decomposition is performed in accordance with the high and low frequency characteristics of a wavelet. This method proposes the following hierarchical threshold estimation.

(9) $$\text{λ}=\delta \sqrt{2\log M*N}*(1-\alpha^{*}j)$$

Where *j* is the resolution scale. *M* × *N* represents image size. 0 < α < 1, and α denotes the adjustment parameter. When we calculate the high-frequency threshold, α is a smaller value, resulting in a slightly larger threshold. When we calculate the low-frequency, α is a larger value, resulting in a slightly smaller threshold. By adjusting α to the threshold parameter α, the accuracy of the threshold estimation is microscopically improved.

## Experiment Analysis

In this paper, the experimental analysis consists mainly of two parts. The objective and subjective evaluation.

### Objective Evaluation

To illustrate the effectiveness of the wavelet threshold algorithm in medical image de-noising, the traditional threshold method and the proposed method was compared. Objective evaluation index is described by peak signal-to-noise ratio (PSNR) and mean square error (MSE).

The PSNR is expressed as follows:

(10) $$PSNR=10*\mathrm{lg}(\frac{255^{2}}{MSE})$$

The MSE is calculated as follows:

(11) $$MSE=\frac{1}{M*N}\left[\sum_{i=1}^{M}\sum_{j=1}^{M}{\left((g(i,j)-\hat{g}(i,j))\right)}^{2}\right]$$

Where *M* ∗ *N* is the size of image; *g*(*i, j*)denotes original image, and ĝ(*i, j*)represents the restoration image. Our data were obtained from the Chinese People's Liberation Army 118 Hospital. The results shown in Table 1 compare the hard threshold method, the soft threshold method and the proposed method.

**Table 1:** De-noising results in different ways of MRI 1.

**MSE**	**0.01**	**0.03**	**0.05**	**0.1**	**PSNR**	**0.01**	**0.03**	**0.05**	**0.1**
Hard threshold	310	603	643	1,495		23.21	20.33	20.05	16.49
Soft threshold	386	765	771	1,627		22.64	19.29	19.26	16.01
Proposed	147	311	341	625		26.46	23.20	23.20	20.17

Through simulation experiments, the data in Tables 1–3 show that the proposed method obtains a large peak signal-to-noise ratio and a smaller mean square error. Thus, our improved wavelet de-noising effect is better.

**Table 2:** De-noising results in different ways of MRI 2.

**MSE**	**0.01**	**0.03**	**0.05**	**0.1**	**PSNR**	**0.01**	**0.03**	**0.05**	**0.1**
Hard threshold	624	780	910	1,136		20.18	19.21	18.54	16.49
Soft threshold	740	952	1080	1,530		22.64	19.29	19.26	16.01
Proposed	206	338	483	668		24.99	22.84	21.29	19.83

**Table 3:** De-noising results in different ways of MRI 3.

**MSE**	**0.01**	**0.03**	**0.05**	**0.1**	**PSNR**	**0.01**	**0.03**	**0.05**	**0.1**
Hard threshold	387	350	549	1,022		22.53	22.67	20.74	17.77
Soft threshold	465	422	647	1,308		21.46	21.88	20.02	16.96
Proposed	180	133	287	631		25.58	26.89	23.55	20.13

### Subjective Evaluation

**Figure 1:**
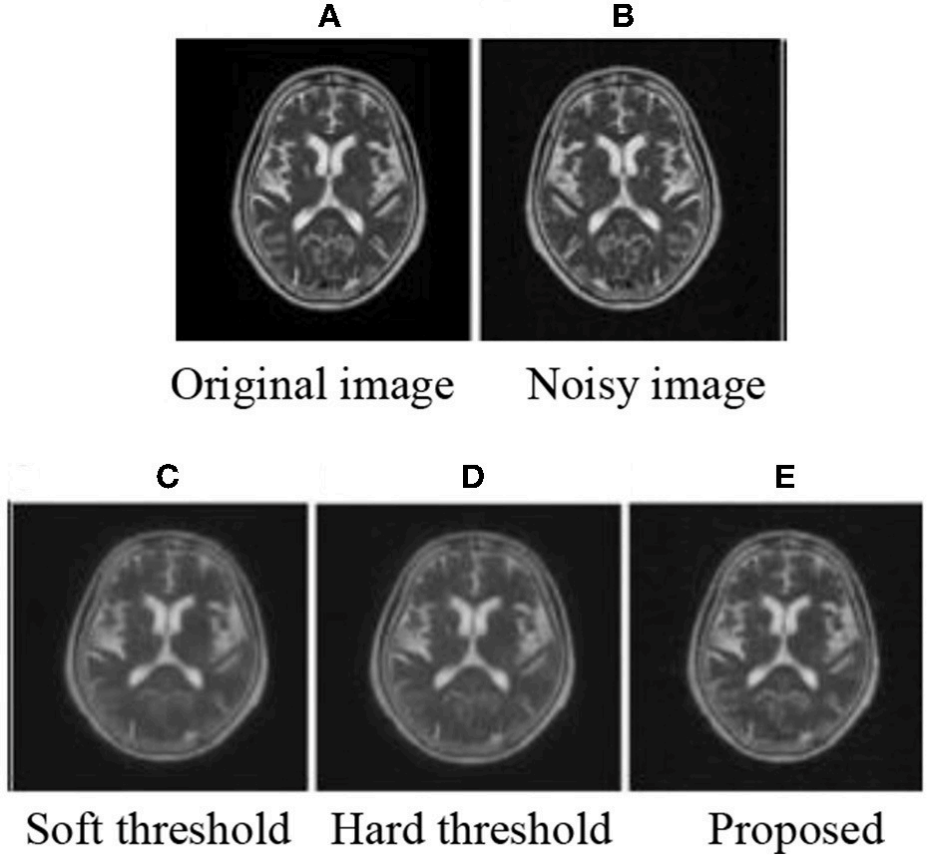
MRI 1 of subjective results.

**Figure 2:**
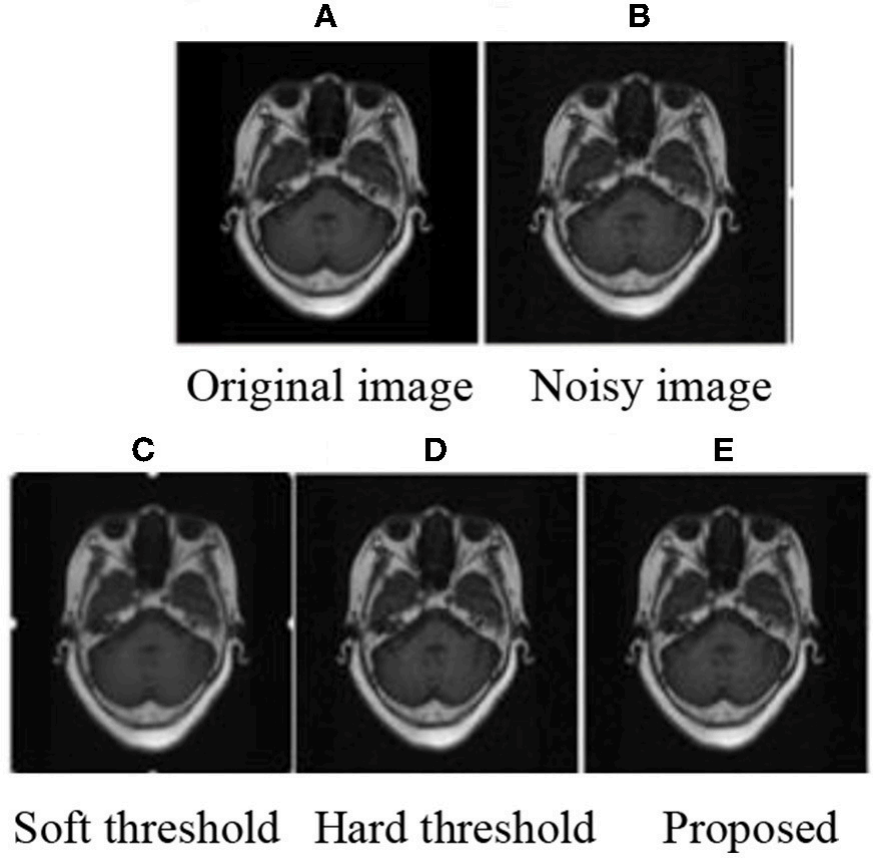
MRI 2 of subjective results.

**Figure 3:**
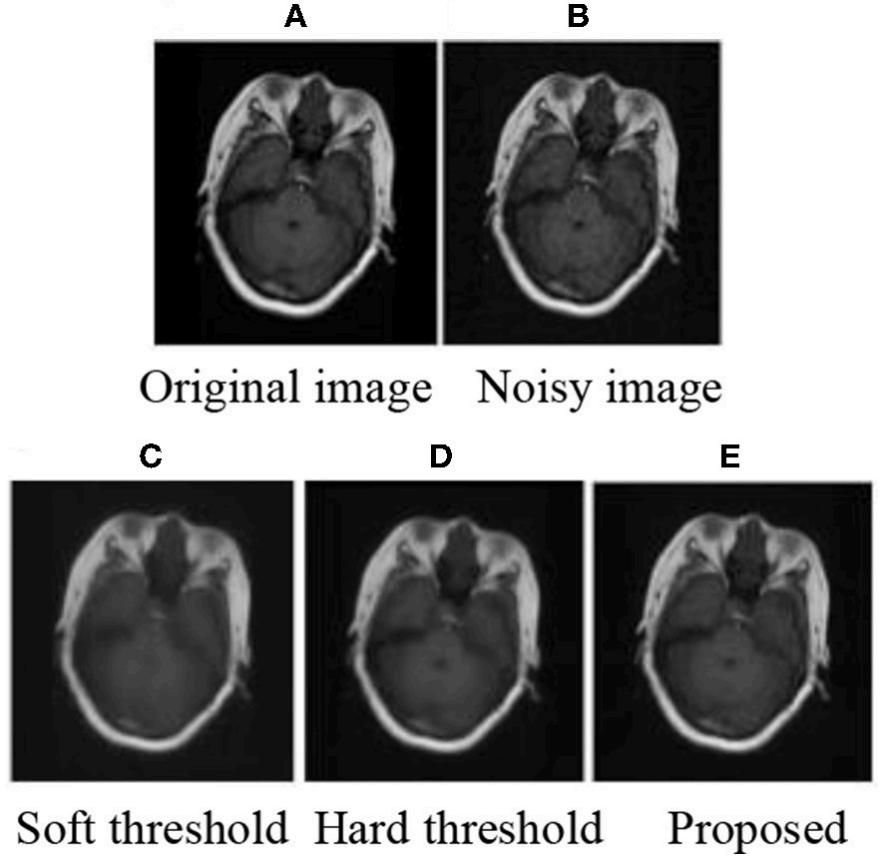
MRI 3 of subjective results.

The experiment was programmed in MATLAB2014 (b). MRI brain images were used to prove the effectiveness of the improved threshold function in medical image de-noising. After decomposition, the threshold was calculated using Equation (9) and processed by the corresponding threshold. Finally, the image was reconstructed to obtain the image after de-noising. The subjective experimental results show that the method proposed in this paper can achieve improved de-noising effects. De-noising effects are achieved when the mean value is 0 and the variance is as follows: 0.01, 0.03, 0.05, and 0.1. The experimental results are shown in Figures 1–3

After adding noise, the original image was almost drowned by noise. Using soft and hard thresholds to remove noise, considerable noises remained in the image. Given the increase in noise, the image appears smoother by using soft and hard thresholds to remove noise. The method in this paper, removed all the noise in the image, and the image was relatively clear. By contrasting the experiments, we suggest that the proposed method has a better effect than hard and soft threshold methods.

## Conclusion

In this study, we analyzed the shortcomings of traditional hard and soft threshold functions for medical image de-noising. We proposed an improved threshold function for de-noising. The mediation factor was increased to find the best estimate of the wavelet coefficient function. The wavelet coefficients were smoothed by the soft threshold function. Thus, the image looks smooth when noise is removed via soft threshold. Through subjective and objective evaluations, the results show that the effect of the hard threshold function is better than that of the soft threshold. However, the signal will produce jumping points when generating additional shocks and the original signal will not be the smooth. The hard threshold method will predict the ringing effect. Improved threshold selection based on the multi-layer wavelet transform, overcomes the disadvantages of soft and hard thresholds. Experimental results showed that the proposed method in this paper can effectively improve the de-noising performance of both soft and hard threshold functions.

## Author Contributions

YZ conceived the study, designed model, and wrote the draft. WD provided data, acquired the pre-processed the data and analyzed the data. ZP and JQ gave critical revision. All authors consent for this submission.

### Conflict of Interest Statement

The authors declare that the research was conducted in the absence of any commercial or financial relationships that could be construed as a potential conflict of interest.
